# The broad spectrum of clinical manifestations observed in three patients with L2 hydroxyglutaric aciduria spans from febrile seizures to complex dystonia

**DOI:** 10.1016/j.ymgmr.2024.101135

**Published:** 2024-08-24

**Authors:** A. Alsayed, M. Albadrani, A. Obaid, A. Alhashim, A. Alakkas

**Affiliations:** a Department of Neurology, National Neuroscience Institute, King Fahad Medical City, Riyadh, Saudi Arabia; b Alfaisal University, College of Medicine, Riyadh, Saudi Arabia; c Department of Pediatric Neurology, National Neuroscience Institute, King Fahad Medical City, Riyadh, Saudi Arabia; d Movement Disorders Division, Department of Neurology, National Neuroscience Institute, King Fahad Medical City, Riyadh, Saudi Arabia

**Keywords:** L2 hydroxyglutaric, Aciduria, Clinical spectrum

## Abstract

L-2 hydroxyglutaric aciduria (L-2-HGA) is a rare autosomal recessive progressive, organic aciduria which presents with a wide variety of clinical manifestations. Diagnosis is complex and necessitates an increase in clinical suspicion of the disease to obtain the necessary diagnostic tests and thus early administration of appropriate management.

In this case series, we are reporting three cases of patients with L-2-HGA who presented with a variety of clinical manifestations. All patients presented with a constellation of symptoms including febrile seizures, hyperactivity and intellectual difficulties. One case had an unusual presentation of cervical dystonia in early adulthood. Another case had a homozygous variant, *L2HGDH*: NM_024884.3: c.368 A > G p. (Tyr123Cys) classified as variant of uncertain significance (VUS) at that time but recently has been reclassified as likely pathogenic variant in clin var. Furthermore, brain MRI of two patients depicted characteristic signs consistent with L-2-HGA. The findings include, symmetrical confluent high T2/FLAIR signal intensity of the white matter involving the subcortical U fibers and deep white matter with sparing of the immediate periventricular white matter, internal capsules and corpus callosum. There was also symmetric abnormal T2 signal intensity of the caudate nuclei, lentiform nucleus as well as the dentate nuclei of the cerebellum.

Overall, only few cases with similar genetic mutation have been documented in the literature and were of Saudi origin. The aim of the study is to highlight the clinico-radiological features of L-2-HGA to aid in early, prompt diagnosis, and thus appropriate follow up and management of the disease with riboflavin, levocarnitine and a low-lysine diet.

## 1 Background

L-2 hydroxyglutaric aciduria (L-2-HGA) is an autosomal recessive progressive, organic aciduria caused by a mutation in L-2 hydroxyglutarate dehydrogenase (*L2HGDH*) gene [1,2]. This results in the deficiency of the mitochondrial enzyme L-2 hydroxyglutarate dehydrogenase [1] resulting in the accumulation of L-2 hydroxyglutarate in tissues leading to the constellation of clinical symptoms [2]. It is postulated that the clinical symptoms are due to the oxidative damage of the intermediary metabolite [3]. There is a broad spectrum of clinical manifestations with CNS predilection [3]. The onset of L-2-HGA is usually in early childhood with epilepsy presenting initially as febrile seizures or psychomotor delay, though later onset of disease has been reported [3,4]. Other presenting symptoms include macrocephaly, features of pyramidal and extrapyramidal dysfunction [4]. Here in, we are aiming to highlight the clinico-radiological features of L-2-HGA to aid in early, prompt diagnosis, and thus appropriate follow up and management of the disease with riboflavin, levocarnitine and a low-lysine diet.

## 2 Case presentation

### 2.1 Case 1

This case concerns a male patient in his twenties who is single and living with his family. He was a full-term twin baby, delivered via caesarean section and had not been admitted to NICU. The mother reported a normal motor developmental history in comparison to his siblings, since he had achieved all the motor milestones, such as sitting, crawling, walking and running, at the same age as his siblings. However, she remarked that he had been considered a hyperactive child. The patient was at elementary school when his teachers alerted the family that he had significant learning difficulties, and he was enrolled in a special needs school for learning disability. His condition remained stable until the age of 19, when the mother noted an inward-turned left foot, and then he started dragging that foot while walking. The family regarded this as a subtle finding and did not seek any medical opinion at that time; however, at the age of 26, the patient started to develop right head-turning with intermittent head tremors, and left head-turning was also noted. The patient also described an infrequent pain at the back of his head and palpitations from time to time. Of note, the mother stated that whenever the patient was febrile or had an illness, he would develop generalised tonic clinic seizures. The mother denied that the patient had any other neurological symptoms, such as weakness, numbness, visual problems and denied frequent falls.

Family history was significant for the twin brother, who had seizures, which were controlled by levetiracetam. Another sibling had learning difficulties, and a third brother was otherwise healthy. When the patient was seen in the clinic, he was alert and followed simple commands with no dysmorphism, microcephaly or organomegaly. His physical exam was significant for right lateralcollis as well as retrocollis with intermittent irregular dystonic head tremors, very slight bilateral kinetic tremors in the upper limbs and generalised exaggerated reflexes (+3), with clonus in the bilateral feet. His gait examination revealed interning of the left foot that improved with backward walking. He had normal power and intact sensation to all modalities. The initial impression was complex multifocal dystonia of unclear aetiology, with neurometabolic disorders being at the top of the differential diagnosis. Patient was initially treated with a combination of trihexyphenidyl, baclofen and Botox injections for cervical dystonia. His work up included the following, Urine organic acid was reviewed, and it was significant for high urinary excretion of 2-hydroxyglutaric acid and 2-oxoglutaric acid. Plasma amino acid was significant for elevated levels of glutamic acid (114.1 umol/L; normal range: 13–113 umol/L), and lysine (422.1 umol/L; normal range 103–255 umol/L). Cento neuro NGS panel was positive for homozygous pathogenic variant c.903 T > G p.(Tyr301*) identified in *L2HGDH* gene. This result was consistent with the genetic diagnosis of autosomal recessive L-2-hydroxyglutaric aciduria. At that point, riboflavin 100 mg daily and folic acid 1 mg daily were added to the patient's treatment regimen. He was referred to genetic clinic to aid in the management plan and to a nutritionist to initiate a low-lysine diet. Later, levocarnitine 1980 mg daily was added to the patient's treatment. Overall, the patient reported improvement after starting riboflavin and folic acid therapy along with levocarnitine, mainly in the form of increased attention. He also reported improvement in neck dystonia and pain, but this was in the setting of ongoing dystonic treatment as noted above.

### 2.2 Case 2

This is a 7-year-old male, who is a product of a full-term uncomplicated pregnancy delivered through spontaneous vaginal delivery and a birth weight of 3.2 kg, with a history of Neonatal intensive care unit admission for 4 days secondary to respiratory distress. He gained all his motor milestones in an appropriate age however he experienced a delay in speech as he was able to speak 2-3 words sentences at the age of 3 years, and only recently was able to tell stories. However, his speech is non-fluent as he displays speech disarticulation and abnormal pronunciation as he replaces some letters with others. Currently the patient is a grade 1 with poor school performance. His parents are from the same tribe, but they are not 1st or 2nd degree cousins, there are two other siblings who are healthy, and has a cousin with intellectual disability and spastic quadriplegia.

At the age of 1 year, the patient developed abnormal movements described as up-rolling of the eyes, with tonic posturing lasting for less than a minute and followed by post-ictal drowsiness. He had in total 3 episodes of seizures happening at yearly interval and all are during a febrile illness. He was seizure free for four years then at the age of 7 he developed a seizure that was not preceded y a febrile illness described as right facial twitching with right sided tonic-clonic movements, at that time he was taken to a local hospital in which he was admitted for work up. He is still experiencing febrile seizures and was started on Levetiracetam 3 50 mg BID, His EEG demonstrated a tendency to develop generalised epilepsy. His parents also noted that he has some intellectual deficits first noticed during school as he is currently repeating grade 1. His examination was remarkable for macrocephaly, no dysmorphic features, hyperactive, fluent speech with mispronunciation, motor examination revealed generalised hypertonia with subtle asymmetry as the right side seems to be involved more. Examination also revealed spasticity and clonus in the right lower limb. Gait examination showed mild tippytoeing with external rotation of the hip, worse on the right side. No tremors were appreciated. IQ assessment was done using the Stanford-Binet intelligence scale and it showed mild impairment or delay in both verbal and non-verbal IQ. His work up consisted of a brain MRI that demonstrated white matter changes in the form of bilateral and symmetrical confluent high T2/FLAIR signal intensity seen involving the subcortical U fibers and deep white matter with sparing of the immediate periventricular white matter, internal capsule and corpus callosum. There is also symmetric abnormal T2 signal intensity of the caudate nuclei, putamina, globe pallidi as well as the dentate nuclei of the cerebellum. Urine organic acids were unremarkable, and whole exome sequencing showed homozygous likely pathogenic variant in *L2HGDH*: NM_024884.2 c.1319C > A p. (Ser440Tyr), confirming the diagnosis of L2 hydroxyglutaric aciduria.

### 2.3 Case 3

This is a 6-year-old male, born to consanguineous parents, who was initially referred to our hospital at 18 months of age due to episodes of atonic seizure. Those episodes started at the age of 1 5 months of age and were mostly provoked by febrile illnesses. They were described by the family as up rolling of eyes with loss of body tone for less than 5 min. The mother reported uneventful pregnancy. His neonatal history was unremarkable, and his growth parameters were within normal limits for age. Furthermore, he had normal development apart from speech delay. His neurological exam was remarkable for hyperactivity, delayed speech, and exaggerated deep tendon reflexes with left sided non sustained clonus. His Social and Emotional Intelligence (EQ) Total Score showed mild deficit of adaptive behaviour with a total score of 59. Due to his distractibility, an official Intelligence quotient (IQ) test could not be performed. To investigate his seizure a brain MRI was done which revealed, bilateral symmetrical confluent high T2/FLAIR signal intensity of the white matter seen involving the subcortical U fibers and deep white matter with sparing of the immediate periventricular white matter, internal capsules and corpus callosum. There was also symmetric abnormal T2 signal intensity of the caudate nuclei, putamina and globi pallidi as well as the dentate nuclei of the cerebellum. His urine organic acid showed significant excretion of 2-hydroxyglutarate. Genetic testing via whole exome sequencing uncovered a homozygous variant, L2HGDH: NM_024884.3: c.368 A > G p. (Tyr123Cys) classified as variant of uncertain significance (VUS) at that time but recently has been reclassified as likely pathogenic variant in clin var. Segregation analysis showed that both parents are carriers for the mutation along with two other siblings. His initial electroencephalogram (EEG) demonstrated generalised intermittent slowing of 2-3 Hz, with medium amplitude. Initially, the patient was not started on antiepileptics as all his seizures were provoked by febrile illnesses. Follow up EEG revealed an epoch of high voltage generalised spikes and poly spike seen lasting for 600 ms followed by slow wave for 1 s, which clinically correlated with eye deviation to the right side. Due to the abnormal EEG and imaging findings, he was started on levetiracetam which resulted in a reduction in seizure frequency, with seizures now only happening during febrile illnesses. He was started on l-carnitine and riboflavin and followed up by a multidisciplinary team. Despite following with speech therapy, behavioural therapy, and genetics. Minimal improvement was reported by the family in terms of speech. His behavioural Symptoms are the most bothering for the family as his distractibility and hyperactivity caused him to fail first grade, therefore requiring special needs schooling.

## 3 Discussion

L-2-HGA was first described by Duran et al., in 1980 [5] since then many other cases have been reported worldwide. There is limited number of studies of L-2-HGA in patients of Arabic ethnicity. The aim of this study is to assess clinical, biochemical, and genetic characteristics of patients with L-2-HGA.

The clinical course of L-2-HGA is usually insidious, however rapid deterioration presenting with acute encephalopathy have been reported [2,6]. In our patients the symptoms were insidious presenting initially as febrile seizures and intellectual decline with prominent behavioural symptoms. Similarly, in a recent cohort of 10 patients with L-2-HGA, the most common symptom was febrile seizures in half of the patients followed by cerebellar symptoms [2]. This highlights the importance of considering the diagnosis of L-2-HGA in patients with febrile seizures and intellectual disability.

In our study however, we encountered an unusual presentation of L-2-HGA presenting with adult-onset multifocal dystonia, including both cervical and foot dystonia, this case presentation consisted of an insidious, subtle onset of cognitive decline and febrile seizures from the first decade of life, which were later exacerbated in early adulthood with the emergence of debilitating cervical dystonia and gait disturbances secondary to foot dystonia. This presentation sheds light on the importance of having high index of suspicion for neurometabolic disorders in an adult presenting with a picture of complex dystonia (dystonia associated with other neurological manifestations beyond movement disorders). Few cases of L-2-HGA with movement disorder manifestations have been reported in the literature. All the cases comprise individuals who initially present with learning disabilities and subsequently manifest movement disorder symptoms as they progress into adulthood [7,8]. This encompasses a diverse array of conditions, with some individuals experiencing tremors while others reported features of dystonia alone or either in isolation or in combination [7,8]. Similar to our case, a 43-year-old female described by Samuraki et al., exhibited signs of both action tremor and multifocal dystonia [7]. Initial treatment with trihexyphenidyl and baclofen did not lead to notable improvement [7]. Subsequently, upon administration of Levocarnitine, the patient displayed marked improvement of dystonic symptoms [7].

The diagnostic approach for L-2-HGA encompasses a combination of biochemical, genomic, and radiological characteristics [9]. The key characteristic of L2HGA is the increased concentrations of L-2-hydroxyglutaric acid detected by biochemical assessment of urine and cerebrospinal fluid (CSF) [1,9] Additionally, there is a potential for a slight increase in plasma levels of L-2-hydroxyglutaric acid [1,9]. The diagnosis of L2HGA or D-2-hydroxyglutaric aciduria (D2HGA), distinct organic acidurias, is determined by the elevated levels of the specific isoform - either the L form or D form - of 2-hydroxyglutaric acid [1]. Hence, additional examination of 2-hydroxyglutaric acid isoforms or genetic testing is necessary for precise diagnosis [1].

To the best of our knowledge, there are around 70 reported genetic mutations of L2HGA gene worldwide [9]. The first patient in this study harboured *L2HGDH*: NM_024884.2:c.903 T > G p.(Tyr301*) which was described initially by Steenweg et al. in 2010 [9]. The second variant identified in the second patient, *L2HGDH*: NM_024884.2:c.1319C > A p. (Ser440Tyr) was identified previously in one Saudi family in 2008 [10]. The same mutation was reported again in 2014 in a Saudi male [11]. To this point, this mutation has solely been documented in Saudi families, which could be a founder mutation; thus, additional research on the different phenotypes is necessary. The third mutation noted in this series, *L2HGDH*: c.368 A > G p. (Tyr123Cys), had been previously documented in a child of Turkish origin [12]. Both our patient and theirs displayed language delay; however, in contrast to our patient, the other individual demonstrated intention tremor, cerebellar ataxia, and macrocephaly [12]. This suggests a poor phenotype genotype correlation with a broad spectrum of clinical manifestations for the same variant as some individuals experience more pronounced symptoms than others.

Disorders causing dystonia represent a heterogenous group of conditions, making diagnosis challenging. The initial step involves identifying the clinical range of symptoms, followed by specific laboratory tests tailored to these clinico-radiological observations [13]. A crucial step in the diagnosis of dystonia is genetic testing. The most common type of genetic testing is by targeted gene panels and in some cases whole exome sequencing. In a study conducted by T Wirth et all, the diagnostic yield of WES in patients with a negative dystonia panel reached 34.4% [13]. The diagnostic outcome was statistically significant for individuals with complex dystonia, particularly when accompanied by intellectual disability [13]. Additionally, individuals exhibiting pyramidal symptoms and patients experiencing dystonia from infancy had a higher rate of successful diagnosis compared to those with later onset [13]. As noted above the confirmatory diagnostic test is gene sequencing whether as part of single gene or gene panel, though L2HGA can present as dystonia unfortunately it is not included in the comprehensive dystonia panels among a lot of accredited commercial molecular genetics lab. We would recommend adding this gene to the comprehensive dystonia panels.

The final crucial diagnostic tool for individuals with this condition is brain MRI. In cases of L2HGA, MRI reveals symmetrical, confluent, and extensive abnormalities in the subcortical white matter, primarily affecting the frontal lobe, with relative preservation of deep periventricular white matter, commissural fibers, and internal capsule [2,3]. Caudate nucleus and lentiform nucleus are prominently affected [4]. In addition, signal abnormalities or atrophy of the dentate nucleus were observed in majority of patients with L2HGA [4]. These radiological findings are considered characteristic for L2HGA but not limited to this condition. Both pediatric patients in this series showed classic MRI findings of L2HGA. Moreover, L2HGA has been connected to the development of central nervous system neoplasms such as medulloblastoma, primitive neuroectodermal tumors, and astrocytoma [14]. Clear guidelines for the treatment and screening of tumors in individuals with L2HGA are yet to be established. Studies have shown that the supplementation with riboflavin and levocarnitine may provide symptomatic improvement [7], while responses to treatment can vary among individuals, as demonstrated in a different study [1].

In the series of cases presented, we emphasized the importance of significance of early identification of the clinical manifestations and imaging characteristics associated with L-2-HGA to prompt the request for the necessary diagnostic evaluations for the condition and thus initiating the management as soon as possible.

### 3.1 Learning points

• L-2-hydroxyglutaric aciduria presents with febrile seizures, mild intellectual disability and can have abnormal movements such as dystonia.
• Physicians should have high index of suspicion for L-2-HGA when encountering patients with complex dystonia to order appropriate genetic testing needed to reach the diagnosis.
• Patients with L-2-hydroxyglutaric aciduria have characteristic MRI images that include symmetrical confluent high T2/FLAIR signal intensity of the white matter involving the subcortical U fibers and deep white matter with sparing of the immediate periventricular white matter, internal capsules and corpus callosum.
• Administration of riboflavin, levocarnitine, and a diet low in lysine for L-2-HGA-associated dystonia results in symptomatic relief in some patients.

## Ethics

Written informed consent was obtained from the patients or their parent to publish this report. Report has obtained an exempt approval by KFMC IRB, IRB log number 24–263.

## Funding

None.

## CRediT authorship contribution statement

**A. Alsayed:** Writing – original draft. **M. Albadrani:** Writing – original draft. **A. Obaid:** Writing – original draft. **A. Alhashim:** Writing – review & editing. **A. Alakkas:** Conceptualization, Writing – review & editing.

## Declaration of competing interest

The authors declare that there is no conflict of interest regarding the publication of this article.

## Data Availability

No data was used for the research described in the article.
